# Report of the General Secretary

**Published:** 1918-01

**Authors:** 


### REPORT OF THE GENERAL SECRETARY

*To the House of Delegates of the National Dental Association:*

The following table shows the organization of constituent societies. It also makes a comparison of the 1917 membership with the 1916 report on membership, by state societies.

| STATE | No. Component Societies | No. Members in 1916 | No. Members in 1917 | No. Delegates 1917 |
|---|---|---|---|---|
| Alabama | .. | 129 | 196 | 2 |
| Alaska Territory | .. | .... | 1 | 1 |
| Arizona | .. | 25 | 24 | 1 |
| Arkansas | .. | 106 | 135 | 2 |
| California State | .. | 406 | 679 | 4 |
| California Southern | .. | 151 | 440 | 3 |
| Colorado | .. | 242 | 295 | 2 |
| Connecticut | .. | 344 | 396 | 3 |
| Delaware | .. | 3 | 3 | 1 |
| Dept. of Interior | .. | 1 | 1 | 1 |
| District of Columbia | .. | 112 | 123 | 2 |
| Florida | .. | 136 | 148 | 2 |
| Foreign Members | .. | 3 | 8 | 1 |
| Georgia | .. | 213 | 212 | 2 |
| Hawaiian Islands | .. | .... | .... | .. |
| Idaho | .. | .... | .... | .. |
| Illinois | 27 | 1675 | 1844 | 11 |
| Indiana | 7 | 727 | 973 | 6 |
| Iowa | 12 | 710 | 900 | 6 |
| Kansas | 9 | 482 | 440 | 3 |
| Kentucky | 8 | 303 | 354 | 3 |
| Louisiana | .. | 66 | 123 | 2 |
| Maine | .. | 129 | 155 | 2 |
| Maryland | .. | 172 | 210 | 2 |
| Massachusetts | 5 | 665 | 215 | 2 |
| Michigan | 10 | 820 | 765 | 5 |
| Minnesota | 7 | 668 | 745 | 5 |
| Mississippi | .. | 134 | 125 | 2 |
| Missouri | 11 | 705 | 804 | 5 |
| Montana | .. | 59 | 114 | 2 |
| National Capital | .. | 27 | 35 | 1 |
| Nebraska | .. | 369 | 411 | 3 |
| Nevada | .. | .... | .... | .. |
| New Hampshire | .. | .... | 24 | 1 |
| New Jersey | 12 | 435 | 569 | 4 |
| New Mexico | .. | 27 | 49 | 1 |
| New York | 9 | 2889 | 1565 | 9 |
| North Carolina | .. | .... | 291 | 2 |
| North Dakota | .. | 127 | 141 | 2 |
| Ohio | 28 | 1289 | 1327 | 8 |
| Oklahoma | 7 | 255 | 297 | 2 |
| Oregon | 7 | 131 | 184 | 2 |
| Pennsylvania | 15 | 802 | 1443 | 8 |
| Philippine Islands | .. | 2 | 2 | 1 |

| STATE | No. Component Societies | No. Members in 1916 | No. Members in 1917 | No. Delegates 1917 |
|---|---|---|---|---|
| Porto Rico | .. | .... | 37 | 1 |
| Rhode Island | .. | 114 | 119 | 2 |
| South Carolina | .. | 14 | 177 | 2 |
| South Dakota | .. | 3 | 175 | 2 |
| Tennessee | .. | 154 | 265 | 2 |
| Texas | .. | 503 | 463 | 3 |
| Utah | .. | 188 | 116 | 2 |
| Vermont | .. | 117 | 85 | 1 |
| Virginia | .. | 168 | 193 | 2 |
| Washington | 8 | 258 | 451 | 3 |
| West Virginia | 6 | 134 | 182 | 2 |
| Wisconsin | 18 | 446 | 695 | 4 |
| Wyoming | .. | 16 | 49 | 1 |
| U. S. Army Dental Corps | .. | 81 | 44 | 1 |
| U. S. Navy Dental Corps | .. | .... | 13 | 1 |

### MEMBERSHIP

The membership of the National Dental Association has had a steady and commendable yearly increase. The secretary's report of 1914 shows a membership of 11,883. The report of 1915, a membership of 14,424. The report of 1916, 18,112. While the secretary's report to October 12, 1917, notwithstanding the increase of dues to $2, or the fact that a great war crisis had to be faced, we are pleased to report an active membership of 19,830. This is a net increase of 1,718 members over the record of 1916.

